# Causes of mortality in cases with extra nodal natural killer/T-cell lymphoma, nasal type: A cohort study

**DOI:** 10.1371/journal.pone.0214860

**Published:** 2019-04-17

**Authors:** Mei Mei, Yingjun Wang, Mingzhi Zhang

**Affiliations:** 1 Department of Oncology, The First Affiliated Hospital of Zhengzhou University, Zhengzhou, Henan Province, China; 2 The Academy of Medical Science, Zhengzhou University, Zhengzhou, Henan Province, China; Brigham and Women's Hospital, UNITED STATES

## Abstract

**Objective:**

Extra nodal natural killer/T-cell lymphoma (ENKTL), nasal type is a rare and highly aggressive type of non-Hodgkin's lymphoma (NHL) commonly presented in the nasal cavity or lymphatic system. However, the common causes of mortality in ENKTL remain unclear. We conducted a retrospective population-based cohort study to elucidate the different causes of mortality in ENKTL and illustrate the main causal and associated risk factors leading to death.

**Methods:**

The study included patients diagnosed with ENKTL from 1987 to 2014 in the Surveillance, Epidemiology, and End Results (SEER) program. Univariate survival analysis was conducted using Kaplan-Meier analysis, and multivariate analyses were performed using Cox proportional hazards regression model. Competing-risks regression model was applied to estimate specific risks associated with mortality.

**Results:**

The analysis demonstrated increased mortality in males and patients diagnosed at older age and higher disease stage. NHL was the most common cause of mortality in patients with ENKTL, accounting for 74.13% of deaths in the cohort, followed by other malignant cancers, heart diseases, and infection. However, NHL-specific death events were fewer in patients diagnosed with advanced disease stage compared with incidences of death by other causes such as disease of heart and infections. Significant difference was seen between patients diagnosed earlier than 2000, who showed a higher probability of dying from NHL, and those diagnosed later, who showed propensity to die from other malignant tumors and infection. No differences were found when comparing sex or age at diagnosis.

**Conclusion:**

The most common cause of mortality in cases with ENKTL-NT is NHL. The female sex, diagnosis at young age and early stage are associated with improved prognosis. Further, the classification of Ann Arbor stage and year of diagnosis can provide references of specific causes of death, which might help decrease the mortality rate.

## Introduction

Extra nodal natural killer/T-cell lymphoma, nasal type (ENKTL-NT) is a comparatively rare and highly invasive type of non-Hodgkin's lymphoma (NHL) that originates from NK cells or T cells and is commonly presented in the nasal cavity or lymphatic system.[1–6] The overall incidence of ENKTL-NT from 2004 to 2009 was reported to be 0.03 in SEER dataset from National Cancer Institute (NCI), USA (per 100,000 population).[6] Further, studies have shown that Epstein-Barr virus (EBV) can be a risk factor in the development of ENKTL-NT,[7–9] leading to a poor outcome with regular relapse, rapid progress, or resistance to treatment. In addition, standard guidelines of therapy for ENKTL patients have not yet been established.[1, 10, 11] Although curative therapies, including radiotherapy, chemotherapy, immunotherapy, and autologous peripheral blood stem cell transplantation (auto-PBSCT), have been around for years; the prognosis of ENKTL patients is disappointing; the overall survival (OS) was reported to be around 42% while the 5-year progression-free survival (PFS) was only 29%.[11, 12] Thus, it is necessary to gain an understanding of the causes and associated risk factors leading to mortality in cases with ENKTL-NT for improving prognosis and curbing the disease progression.

Bluhm reported that the causes of mortality in cases diagnosed with NHL were NHL, leukemia, other malignant tumors, cardiovascular diseases and infections.[13] Further, organ transplantations, HIV infections and immunodeficiency syndromes have been reported as other risk factors leading to death.[14] However, the cause of death in cases with ENKTL-NT remains unclear. Therefore, this study aimed at conducting a retrospective population-based cohort study to analyze the various causes of death and risk factors associated with ENKTL-NT and estimated the most common causes, in order to provide references for clinical practice and early prognosis.

## Materials and methods

### Surveillance, Epidemiology, and End Results (SEER) Program

Information regarding the participants was downloaded from the Surveillance, Epidemiology, and End Results (SEER) Program [15], which was carried out by the National Cancer Institute (NCI), USA. The program included 18 registries and covered over 28% of the US population. We identified patients who were diagnosed with ENKTL-NT from 1987 to 2014 in SEER program. The data were selected and analyzed through the SEER*stat software version 8.3.4 (www.seer.cancer.gov/seerstat). SEER is supported by the Surveillance Research Program (SRP) in NCI's Division of Cancer Control and Population Sciences (DCCPS) (www.seer.cancer.gov).

### Patients

Cases were selected based on the classification of lymphoid neoplasms published by International Lymphoma Epidemiology Consortium (InterLymph) Pathology Working Group, according to the WHO classification of lymphoid neoplasms and the International Classification of Diseases-Oncology, Third Edition (ICD-O-3). Cases diagnosed with ENKTL-NT with adequate available information were selected for this study. No restricting criteria were added for age, sex, and race. A total of 163 patients participated in the program from diagnosis till the end of a 5-year follow-up or death, whichever occurred first. However, since we aimed to analyze the cause of death, all patients who survived past the follow-up period were excluded from our study. In addition, all patients with death certificate only or autopsy only were excluded, as were patients whose survival time could not be ascertained. The patients included were those diagnosed with ENKTL as the first primary tumor. The study focused on analyzing the primary cause leading to death. Finally, based on our inclusion and exclusion criteria, 107 patients were analyzed for the cause of death and divided into two groups: NHL-specific mortality and mortality by other causes. The latter was further classified in four sub-groups: (1) other malignant tumors, (2) diseases of heart, (3) infections, and (4) other causes.

### Variables

Variables analyzed included sex (male or female), calendar year of diagnosis (1987–2000, 2001–2007, or 2008–2014), race (Caucasian, African American, others), Ann Arbor Stage of the disease (stage I and II or stage III and IV) and age at diagnosis (< 60 or ≥ 60).

### Statistical analysis

Distributions of survival time were assessed grouped by basic information and Ann Arbor stage of disease in patients with ENKTL, nasal type. The ICD-10 codes for different causes of death were used in the study to standardize the analysis. The overall survival (OS) was defined as the date from diagnosis to the date of death or the end of the follow-up.

Descriptive statistics, including median and confidence intervals (CI), were provided for continuous variables. Frequencies and percentages were used to summarize categorical variables. For comparisons of OS between subgroups classified by sex, Ann Arbor Stage, race and age at diagnosis of patients, survival curves were drawn using the Kaplan-Meier method (Graphpad Prism 6 Software, USA). Univariate analyses were performed by the log-rank test while multivariate analyses were performed using the Cox proportional hazards regression model. The pie nest chart was drawn using Echarts (https://ecomfe.github.io/echarts-doc/public/en/index.html). All statistical analyses were performed using IBM SPSS Statistics for Windows, version 21.

Owing to the nature of retrospective studies, a potential confounding bias may exist. Therefore, the competing-risks regression model was applied using software STATA (version 15) to analyze the competing events. When calculating NHL-specific mortality, death by NHL was the event of interest and other-causes mortality was the competing event, and vice versa. Similarly, when considering the sub-group ‘other causes,’ the four groups were competing events against each other. The analysis of each cause was further grouped by sex, Ann Arbor Stage, calendar year of diagnosis and age at diagnosis of patients. The cause-specific sub-hazard ratios (SHRs) and cumulative incidence function (CIF) were calculated to demonstrate competing risks. We calculated the CIFs for stage, age group, and calendar year of diagnosis. All statistically significant differences were defined as p < 0.05.

## Results

### Distributions of patients

This study included 163 participants with adequate information during the 5-year follow up from the SEER database. Among these participants, 116 died at a median age of 58, with male and female patients accounting for 67% and 33% of the deaths, respectively. The number of patients diagnosed in early years was much smaller than that of patients diagnosed in recent years. Since the data was collected in the U.S., the cohort mostly comprised of White Americans. All patients were classified by the Ann Arbor staging system used for classifying lymphomas (1983+). The number of patients in stage I and II (n = 111, 68.1%) were more than twice compared with that in the advanced stage III and IV (n = 43, 26.38%). Detailed distributions of all cases classified by sex, race, stage, calendar year of diagnosis, and age at diagnosis are presented in Table 1.

**Table 1:** Distributions of characteristics of patients with extra nodal natural killer/T-cell lymphoma, nasal type.

Characteristics	No.	(%)	MS (months)	CI (months)	P-value
Sex					
Male	109	(66.87)	11	(6.288, 15.712)	**P = 0.038**
Female	54	(33.13)	15	(0.000, 36.604)	
Race					
Caucasian	98	(60.12)	12	(4.062, 19.938)	P = 0.100
African American	9	(5.52)	5	(2.078, 7.922)	
Others	56	(34.36)	14	(3.523, 24.477)	
Ann Arbor Stage					
I and II	111	(68.10)	23	(9.499, 36.501)	**P<0.001**
III and IV	43	(26.38)	6	(3.247, 8.753)	
Unknown	9	(5.52)	11	(1.298, 20.702)	
Age at diagnosis					
<60	96	(58.90)	23	(10.782, 35.218)	**P<0.001**
≥60	67	(41.10)	7	(5.234, 8.766)	
Calendar year of diagnosis					
1987–2000	29	(17.79)	28	(0.000, 68.436)	-
2001–2007	70	(42.94)	23	(9.340, 36.660)	
2008–2014	64	(39.26)	-	-	

MS: Median Survival Time, CI: confidence intervals.

### Survival analysis

**Fig 1:**
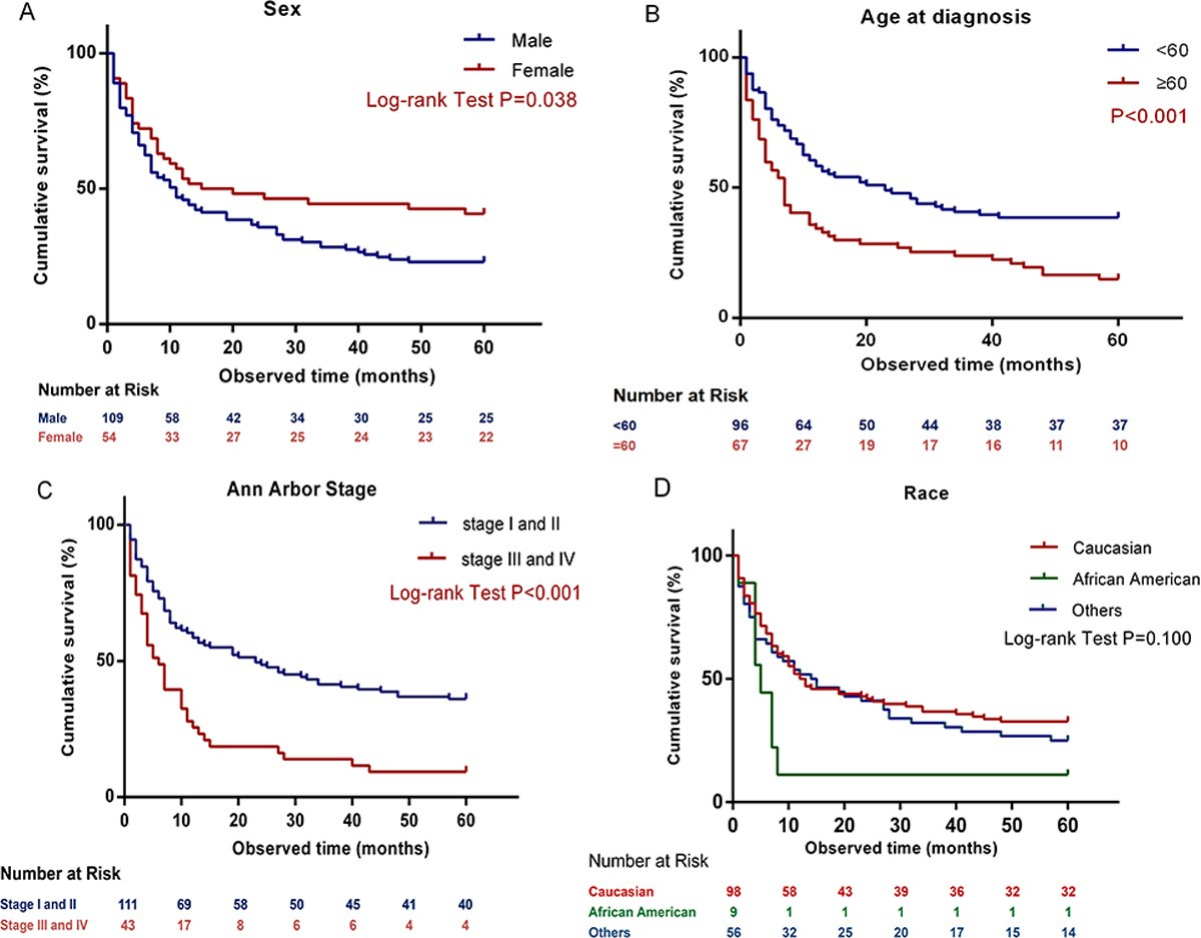
Kaplan-Meier curves for comparison of patients diagnosed with extra nodal natural killer/T-cell lymphoma, nasal type according to (A) Sex, (B) Age at diagnosis, (C) Ann Arbor Stage and (D) Race.

Kaplan-Meier analysis of the cohort demonstrated increased mortality in males, and diagnosis at older age and with higher disease stage. Female patients showed better OS, with median survival time of 15 months, compared with males, who showed a median survival time of 11 months (*P* = 0.038; Fig 1A). The OS rate was higher in the < 60-year-old age group compared with that in the older age group (*P* < 0.001). The median survival duration was 23 months (for patients < 60-year-old) and 7 months (for patients ≥ 60-year-old) (Fig 1B). Further, the patients in stage III and IV displayed a shorter lifetime and higher mortality (*P* < 0.001; Fig 1C). Caucasian Americans and others, including American Indian-Alaska Natives, Asians, and Pacific Islanders showed better OS, with a median of 12 months or 14 months respectively, compared with African American patients, who had a median survival time of 5 (*p* = 0.100; Fig 1D). However, this could be attributable to the small sample size for African Americans compared with that of other races.

The survival rates for 2-year, 3-year and 10-year follow up were 39.9%, 33.7%, and 28.8%, respectively (Table 2). The survival rate dropped quickly in males, other races (including American Indian-Alaska Native, Asian and Pacific Islander), and patients with older age.

**Table 2:** Survival of 2-, 3- and 5-year follow-up in patients with extra nodal natural killer/T-cell lymphoma, nasal type in 1987–2014.

Characteristics	Total number	2-year survival		3-year survival			5-year survival	
		Frequency	Rate	Frequency		Rate	Frequency	Rate
Sex								
Male	109	39	35.8	31		28.4	25	22.9
Female	54	26	48.1	24		44.4	22	40.7
Race								
Caucasian	98	41	41.8	36		36.7	32	32.7
African American	9	1	11.1	1		11.1	1	11.1
Others	56	23	41.1	18		32.1	14	25.0
Ann Arbor Stage								
I and II	111	54	48.6	46		41.4	40	36.0
III and IV	43	8	18.6	6		14.0	4	9.3
Unknown	9	3	33.3	3		33.3	3	33.3
Age at diagnosis								
<60	96	46	47.9	39		40.6	37	38.5
≥60	67	19	28.4	16		23.9	10	14.9
Total	163	65	39.9	55		33.7	47	28.8

Consistently, Cox proportional hazards regression showed that diagnosis at older age (*P* < 0.001) was associated with worse survival (Table 3). In addition, African Americans were associated with worse survival, compared with Caucasians. However, there was no significant difference between sex and stage.

**Table 3:** Hazard ratios and 95% confidence intervals of Cox regression for Mortality Among Patients With extra nodal natural killer/T-cell lymphoma, nasal type Diagnosed Among US Veterans From 1987 to 2014.

Characteristics	Hazard Ratios	P	95.0% confidence intervals	
Sex				
Male	1.00 (reference)			
Female	1.476	**0.075**	0.962	2.264
Race				
Caucasian	1.00 (reference)			
African American	0.455	**0.045**	0.211	0.984
Others	0.575	0.170	0.261	1.267
Ann Arbor Stage				
I and II	1.00 (reference)			
III and IV	0.943	0.893	0.397	2.237
Unknown	1.843	0.183	0.750	4.527
Age at diagnosis				
<60	1.00 (reference)			
≥60	0.502	**<0.001**	0.345	0.731

### Cause of death

Among all the causes listed in Table 4, NHL was the most common cause of death, accounting for 74.13% of all deaths, followed by other malignant cancers. Several of the deaths were also accounted for by infectious and parasitic diseases and diseases of the heart. Apart from these, chronic obstructive pulmonary disease and allied conditions, complications of pregnancy, childbirth, puerperium, congenital anomalies, accidents, suicide, and self-Inflicted injury were also listed as causes of mortality, at a relatively low rate.

**Table 4:** Distribution of causes of death of follow-up in patients with extra nodal natural killer/T-cell lymphoma, nasal type, 1987–2014.

Mortality status	No.	(%)
Total	163	(100.00)
Alive throughout 5-year follow-up	47	(28.83)
Death	116	(71.17)
Non-Hodgkin lymphoma	86	(52.76)
Other malignant cancers	6	(3.68)
Nasopharynx	1	(0.61)
Nose, nasal cavity and middle ear	1	(0.61)
Lung and bronchus	1	(0.61)
Myeloma	1	(0.61)
Miscellaneous malignant cancer	2	(1.23)
Pneumonia and influenza	1	(0.61)
Septicemia	1	(0.61)
Other infectious and parasitic diseases including HIV	5	(3.07)
Diseases of heart	4	(2.45)
Chronic obstructive pulmonary disease and allied cond	1	(0.61)
Complications of pregnancy, childbirth, puerperium	1	(0.61)
Congenital anomalies	1	(0.61)
Accidents and adverse effects	1	(0.61)
Suicide and self-Inflicted injury	1	(0.61)
Other cause of death	5	(3.07)
State DC not available or state DC available but no COD	3	(1.84)

DC: death certificate. COD: cause of death.

### Competing risk regression

Ordinary Cox regression may give possibly biased estimates of cause-specific risk of death by failing to account for competing causes. [15] Therefore, we conducted competing risk regression for cause-specific purpose. The regression model to show the CIF for competing specific risks was first proposed by Fine and Gray and developed further by many researches in various statistical analysis software.[16, 17] The different causes of death were competing events in this study, and a series of regression models were utilized for comparison and risk determination.

Although this study included 116 participants who died within the follow-up period, six of them did not have records on Ann Arbor Stage and three did not have State DC (death certificate) or COD (cause of death). Only 107 had adequate information on COD, sex, Ann Arbor Stage, age at diagnosis, and year of diagnosis. Since all death cases in the African American group could be attributable to NHL, and not to any other cause, we considered these samples to be inadequate to conduct competing risk method analysis.

**Fig 2:**
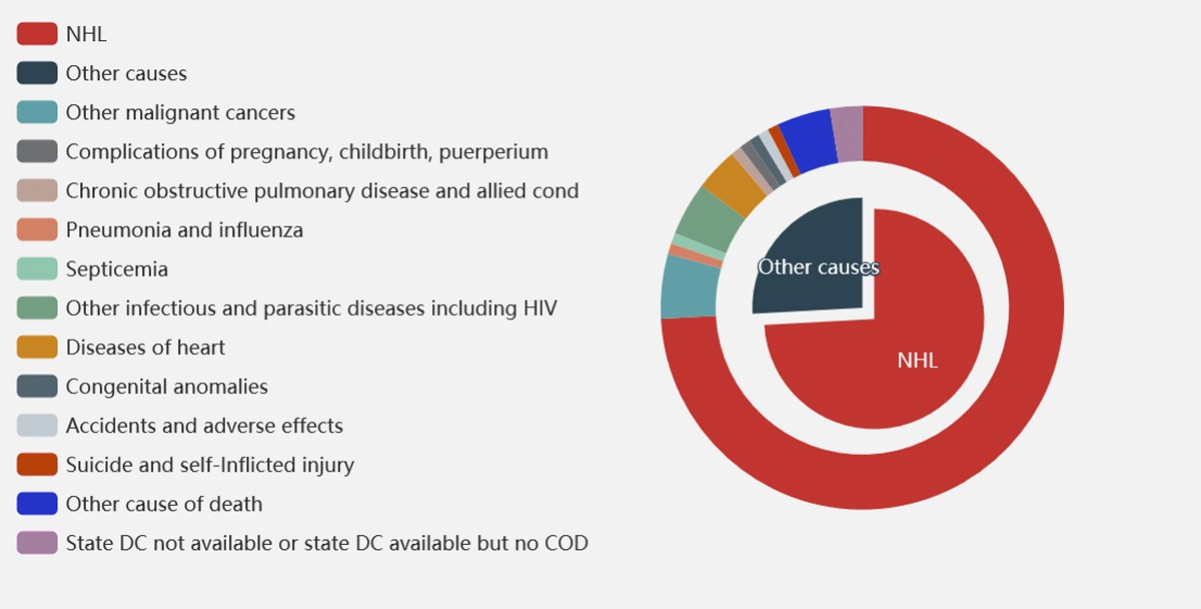
Distribution of causes of death of follow-up in patients with extra nodal natural killer/T-cell lymphoma, nasal type, 1987–2014.

Tables 5 and 6 show the distribution of the 107 cases based on the cause of mortality. Results of the competing-risks regression model demonstrated that for patients of stage III and IV, NHL-specific death events were much fewer, while incidence of death by other causes was higher, such as by disease of heart and infections (Table 6). Significant difference was seen between the patients diagnosed earlier than 2000, who showed a higher probability to die from NHL (Fig 2), compared with those diagnosed at a later calendar year, who showed a higher rate of mortality from other malignant tumors and infection. We found no differences when comparing the sex or age at diagnosis.

**Table 5:** Sub-hazard ratios of cause-specific death by sex, Ann Arbor Stage, calendar year of diagnosis and age at diagnosis by competing-risks regression.

Characteristic	NHL-specific mortality			Other-cause mortality		
	SHR	(95% CI)	P-value	SHR	(95% CI)	P-value
Sex						
Male	1.00(reference)			1.00(reference)		
Female	0.824	(0.275, 2.471)	0.729	0.975	(0.615, 1.547)	0.916
Ann Arbor Stage						
I and II	1.00(reference)			1.00(reference)		
III and IV	0.714	(0.520, 0.980)	**0.037**	1.281	(1.117, 1.468)	**0.001**
Calendar year of diagnosis						
1987–2000	1.00(reference)			1.00(reference)		
2001–2008	0.197	(0.073, 0.532)	**0.001**	3.104	(1.384, 6.960)	0.006
2009–2014	0.481	(0.200, 1.156)	0.102	1.961	(0.875, 4.394)	0.102
Age at diagnosis						
<60	1.00(reference)			1.00(reference)		
≥60	0.757	(0.496, 1.157)	0.199	1.308	(1.033, 1.656)	**0.026**

NHL: non-Hodgkin’s lymphoma SHR: Sub-hazard ratios. CI: confidence intervals.

**Table 6:** Sub-hazard ratios with 95% confidence intervals of other causes rather than non-Hodgkin’s lymphoma by sex, Ann Arbor Stage, calendar year of diagnosis and age at diagnosis by competing-risks regression.

Characteristic	Other malignant tumors		Diseases of heart		Infections		Other causes	
	SHR (95% CI)	P-value	SHR (95% CI)	P-value	SHR (95% CI)	P-value	SHR (95% CI)	P-value
Sex								
Male	1.00 (reference)		1.00 (reference)		1.00 (reference)		1.00 (reference)	
Female	1.440 (0.964, 2.151)	0.075	1.205 (0.799, 1.818)	0.374	0.913 (0.586, 1.423)	0.688	0.920 (0.592, 1.430)	0.711
Ann Arbor Stage								
I and II	1.00 (reference)		1.00 (reference)		1.00 (reference)		1.00 (reference)	
III and IV	1.140 (0.996, 1.305)	0.058	1.217 (1.075, 1.378)	**0.002**	1.230 (1.089, 1.388)	**0.001**	1.224 (1.089, 1.421)	**0.001**
Calendar year of diagnosis								
1987–2000	1.00 (reference)		1.00 (reference)		1.00 (reference)		1.00 (reference)	
2001–2008	1.657 (1.025, 2.680)	**0.039**	1.866 (0.974, 3.577)	0.060	2.962 (1.484, 5.914)	**0.002**	1.566 (0.891, 2.754)	0.119
2009–2014	1.244 (0.720, 2.150)	0.434	1.886 (0.981, 3.627)	0.057	2.437 (1.218, 4.878)	**0.012**	1.279 (0.694, 2.359)	0.430
Age								
<60	1.00 (reference)		1.00 (reference)		1.00 (reference)		1.00 (reference)	
≥60	0.941(0.757, 1.170)	0.583	0.958 (0.774, 1.185)	0.690	1.184 (0.962, 1.456)	0.111	1.197 (0.965, 1.484)	0.102

SHR: Sub-hazard ratios. CI: confidence intervals.

**Fig 3:**
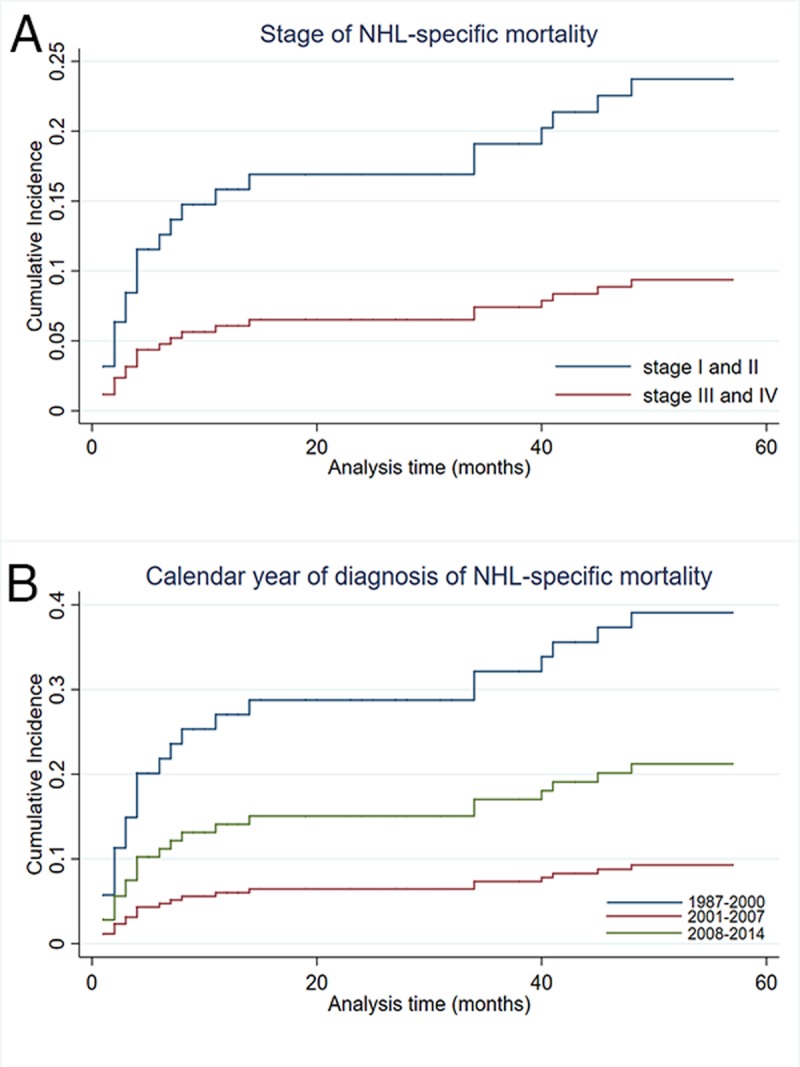
**Cumulative incidence plot** comparing (A) Ann Arbor stage and (B) calendar year of diagnosis in NHL-specific cause mortality.

**Fig 4:**
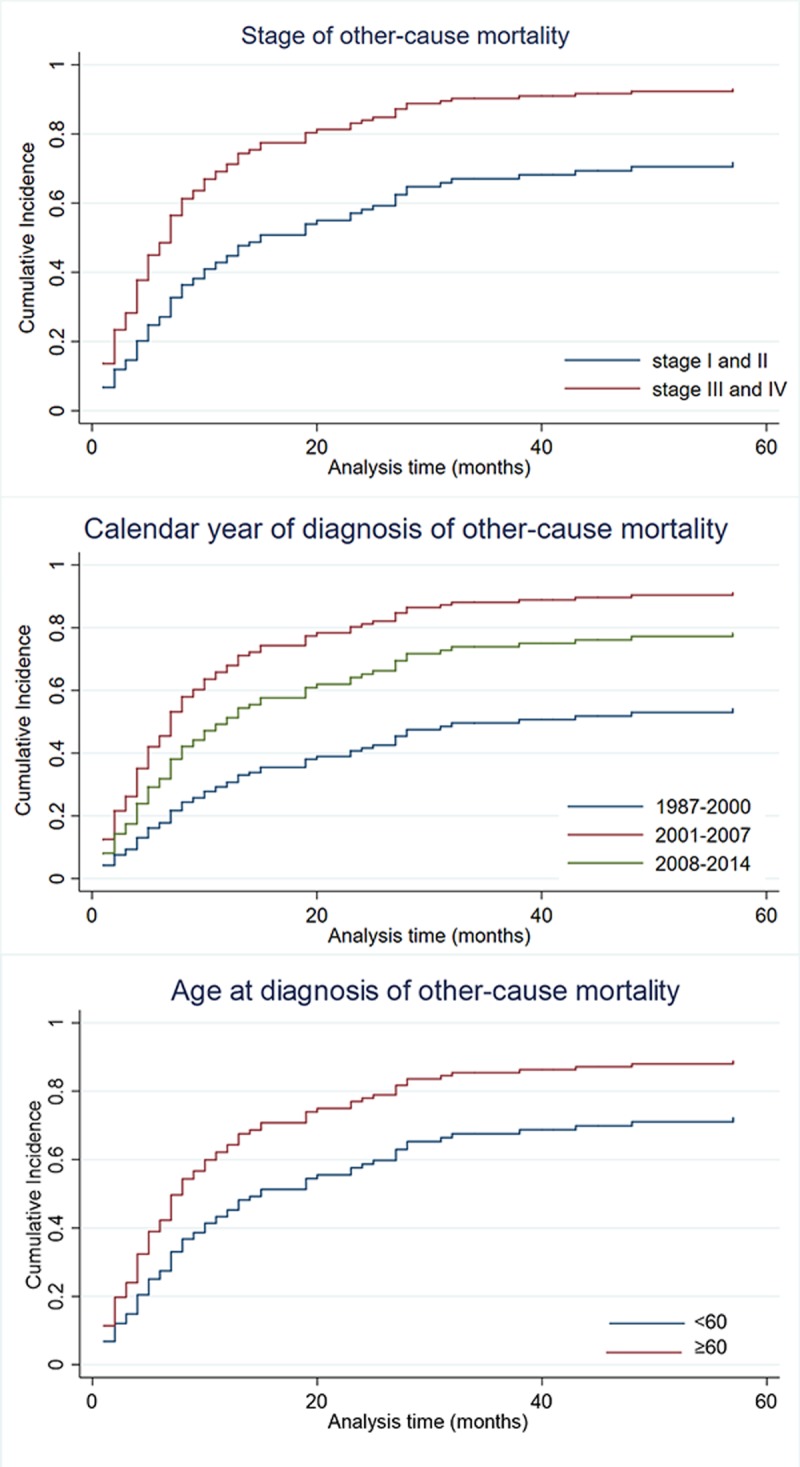
**Cumulative incidence plot** comparing (A) Ann Arbor stage, (B) calendar year of diagnosis (C) age at diagnosis in other cause mortality.

**Fig 5:**
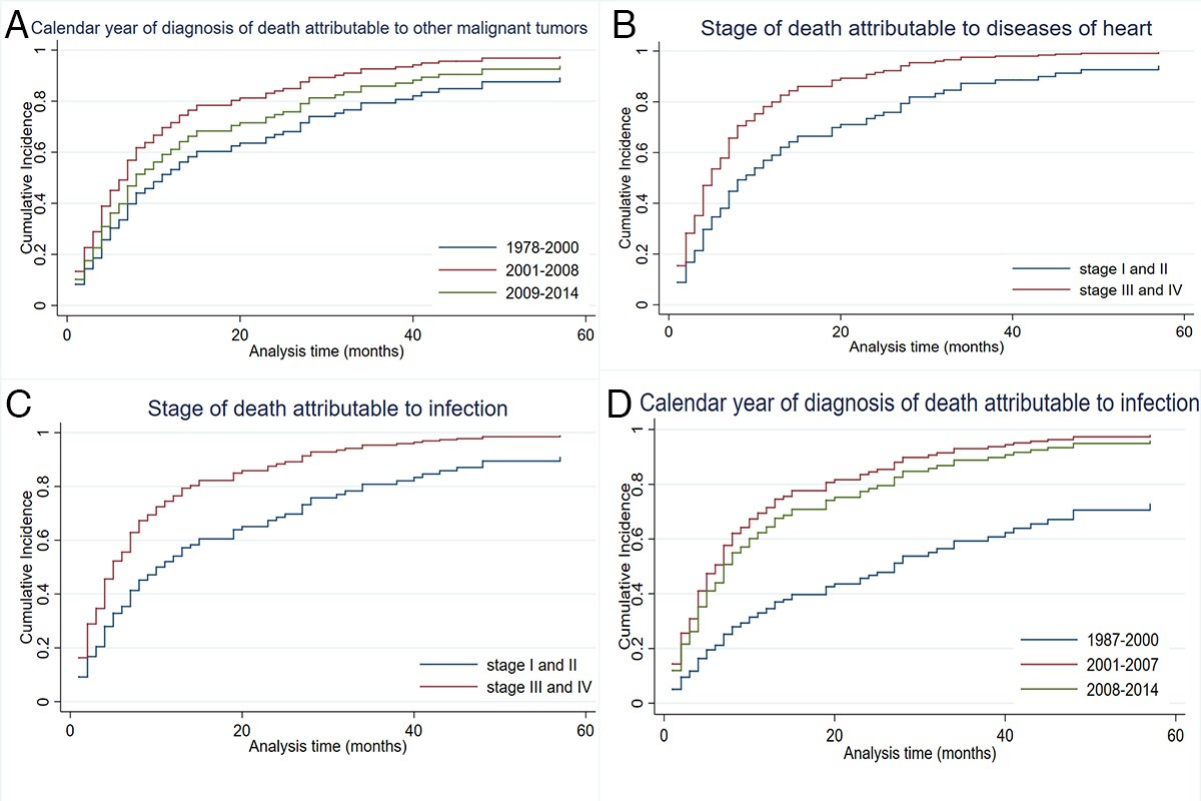
**Cumulative incidence plot** comparing (A) calendar year of diagnosis attribute to other tumors, (B) Ann Arbor stage of death attribute to disease of heart, (C) Ann Arbor stage of death attribute to infection and (D) calendar year of diagnosis attribute to infection.

Figs 3 and 4 present cumulative incidence curves for NHL-specific mortality and other causes of mortality over the 5-year follow-up based on age at diagnosis, stage, and calendar year. In accordance with sub-hazard ratios of cause-specific death by competing-risks regression, the cumulative incidence of NHL-specific mortality increased more rapidly in cases diagnosed at an early calendar year and stage I and II. The cumulative incidences of other causes of mortality (Fig 5) showed the opposite result. In addition, the cumulative incidence of older age was higher in other-cause mortality.

## Discussion

Extra nodal natural killer/T-cell lymphoma, nasal type (ENKTL-NT), is a comparatively infrequent and highly aggressive lymphoma that currently lacks optimal treatment and shows poor outcome. [18] In our study, female patients, diagnosed at younger age and early stage showed a longer survival time than that reported in previous studies. [19–21]Some studies have illustrated that females have a better survival rate in patients diagnosed with ENKTL-NT, consistent with other NHL,[22–24] probably because male patients are more likely to suffer cardiovascular diseases after being diagnosed with NHL[3] and are more likely to use alcohol and tobacco that increase the risk.[25] Advanced age (defined as > 60 years) has been included in the International Prognostic Index as an unfavorable factor for ENKTL-NT disease outcome.[18, 26] Ann Arbor stage is another important prognostic factor in NHL.[18, 22] Patients in advanced stages may receive sequential and multi-cycle chemotherapy or combined radiotherapy that might bring about severe drug toxicity.[18] Indeed, toxic deaths (63%) have been reported, mostly occurring after the first cycle of chemotherapy.[27] Patients in stage IV face the higher risk of acquiring natural killer/T-cell lymphoma-associated hemophagocytic syndrome (HPS),[28] which is scarce, but life-threatening.[29, 30] Moreover, tumor size, which was excluded in staging, also significantly influences prognosis of the disease.[31] In addition, some researchers claim that intra-cavity disease had a better prognosis than extra-cavity disease.[6, 22]

The current retrospective cohort study illustrated that although the most common cause of death with ENKTL-NT was NHL, there is considerable risk of other malignant cancers, including the cancers of nasopharynx, nose, nasal cavity and middle ear, lung and bronchus, myeloma and miscellaneous malignant cancer. Additionally, several factors, including age, sex, stage and time of diagnosis, could be risk factors affecting cause-specific mortality.

The competing-risks regression model was applied in this study to evaluate the relationship of variables to cause-specific failures, [15] while the cause-specific sub-distribution hazard function was applied to indicate cumulative probability (incidence) of the causes.

Death rate attributed to causes other than NHL was much higher for patients in advanced stage of the disease. This could be because patients in the advanced stage are more likely to face aggressive therapies, leading to potential risk of hematologic toxicity. For instance, SMILE (dexamethasone, methotrexate, ifosfamide, L-asparaginase, and etoposide) regimen, which is the current standard to treat advanced-stage ENKTL, may be associated with more severe hematological toxicity compared with other L-asparaginase combinations and gemcitabine-based or CCRT (concurrent chemoradiotherapy) based regimens. [32] SMILE, which leads to the most toxic hematological outcome, has been associated with grades 3–4 neutropenia in 72% of treated patients. [32] After SMILE treatment, 45% patients had grade 3 and 16% patients had grade 4 infection, including 2 patients who died as a result of infection.[33]

Patients diagnosed in this century showed a higher survival rate than those diagnosed in the last century, partly attributed to the standardization of suitable and effective therapies, the development of HSCT,[34] and immune- and cellular therapy.[35] Patients of advanced age (defined as > 60 years) were more likely to face causes other than NHL, because other effects of aging such as a decrease in the baseline cardiac, immune, renal, and hepatic function. [3] No differences were found in variables of sex and age at diagnosis during the cause-specific analysis. However, previous studies have reported that female sex was associated with risks of mortality other than NHL among childhood NHL survivors.[13, 36] This risk factor remains to be further validated in case of ENKTL-NT.

The current study has some limitations. First, some confounding factors may be included due to the retrospective nature of the study. Since we evaluated only the data collected from SEER, other potential risk factors may be missed. Second, a selection bias exists since patients with incomplete information were excluded. Third, there was a lack of consistency in death certificates obtained from different registries. In addition, since ENKTL-NT is a rare subtype of lymphoma, it is difficult to get larger sample pool to decrease the sampling error.

Currently, IPI (patient age, tumor stage, serum lactate dehydrogenase [LDH] concentration, performance status, and number of extranodal disease sites) has been most widely used in NHL prognosis since more than twenty years. [37] However, IPI cannot provide context regarding the patient’s cancer-specific mortality risks in the presence of competing risks. [38] Predicting ENKTL-NT outcome and stratifying patients for different competing risk events are a challenge for physicians, making it important and urgent to identify novel biomarkers for better prognosis.

## Conclusion

Our study indicates that the female sex, diagnosis at young age and with early stage of the disease are factors associated with better prognosis for ENKTL-NT. This population-based cohort study revealed that the most common cause of mortality in cases with ENKTL-NT is NHL, rather than other malignant cancers, diseases of heart, infections or other causes. Our study emphasizes that more effort should be made to identify patients in early stages so as to implement comprehensive treatment and prevent the occurrence of high-risk factors. Furthermore, the Ann Arbor staging and calendar year of diagnosis provide references for cause-specific death, and might help during the clinical procedures to decrease the overall and cause-specific mortality rate.

### Availability of data and materials

The datasets analyzed are available in the Surveillance, Epidemiology, and End Results (SEER) program (www.seer.cancer.gov) which is supported by the Surveillance Research Program (SRP) in NCI's Division of Cancer Control and Population Sciences (DCCPS).Because of the sensitive nature of the data, SEER Data Use Agreement (https://seer.cancer.gov/seertrack/data/request/) is requested for everyone before accessing to the database through SEER*Stat's Client-Server Mode (https://seer.cancer.gov/seerstat/).

### Ethics approval and consent to participate

Since the database used was a group of public sets, it was exempted from institutional review boards. The participants were requested using the National Cancer Institute SEER*stat software version 8.3.4 (www.seer.cancer.gov/seerstat).
